# A Novel Interstitial Site in Binary Rock-Salt Compounds

**DOI:** 10.3390/ma15176015

**Published:** 2022-08-31

**Authors:** Neeraj Mishra, Guy Makov

**Affiliations:** Department of Materials Engineering, Ben-Gurion University of the Negev, Beer Sheva 84105, Israel

**Keywords:** rocksalt, interstitials, defect energetics, electronic structure

## Abstract

The energetic and mechanical stability of interstitial point defects in binary rock-salt materials were studied using the first-principles method. A novel, stable, and energetically competitive interstitial site (base-interstitial) was identified for anion interstitials in rock-salts. The formation energies of base-interstitial defects were compared with well-explored tetrahedral (body-interstitial) and split interstitials and were found to be energetically highly competitive. For alkali halides and silver bromide, the lowest formation energies are associated with the base-interstitial site and the <110> split interstitial, which are therefore the predominant interstitial sites. However, split interstitials were found to be the energetically preferred configuration in metal monochalcogenide systems. Electronic band structures are affected by the presence of interstitial defects in rock-salt structures. In particular, the Fermi level is shifted below the valence band maxima for the body, base, and split interstitials in metal halides, indicating p-type conductivity. However, the Fermi level remains within the bandgap for metal monochalcogenides, indicating no preferred conductivity for base- and split-interstitial defects. Allowing the defects to be charged changes the relative stability of the interstitial sites. However, the new base-interstitial site remains preferred over a range of potentials for alkali halides. The anion base-interstitial is found to form a triatomic entity with the nearest lattice anions that affect the electronic structure relative to the body interstitial. The discovery of a new interstitial site affects our understanding of defects in binary rock-salts, including structure and dynamics as well as associated thermodynamic and kinetic properties that are interstitial dependent.

## 1. Introduction

Point defects in materials lead to structural, electrical, and mechanical changes, which can be detrimental in some applications [1], e.g., point defects can affect energy storage capacity [2,3], catalyze chemical reactions [2], control light emission efficiency [4,5], and tune the thermal [6,7,8] and electrical [9,10] properties of materials. Point defects have finite formation energies and are established as equilibrium species in a crystal. However, semiconductor materials used in devices, such as communication equipment, satellites and detectors, undergo radiation damage by electrons, particles and photons [11]. Such high-energy particles can cause the formation of point defects in the crystal lattices of semiconductors. Moreover, such irradiations may change the concentration of equilibrium point defects formed during growth [11]. The stability of a point defect configuration in a material provides information about its feasibility and equilibrium concentration.

Rock-salt structures are an important cubic structure type with space group $Fm\bar{3}m$, of which NaCl is a well-known example. Rock-salt structures are generated when anions form a face-centered cubic (FCC) unit cell with octahedral and tetrahedral interstitial sites. The cations occupy all octahedral sites and leave tetrahedral sites vacant [12]. Therefore, each anion is octahedrally coordinated by six cations; similarly, each cation is coordinated by six anions in a rock-salt structure. Many AB compounds possess a rock-salt structure. Most hydrides and halides of alkali metals and Ag^+^, as well as a large number of chalcogens (oxides, sulfides, etc.) [12], also possess this structure.

**Figure 1:**
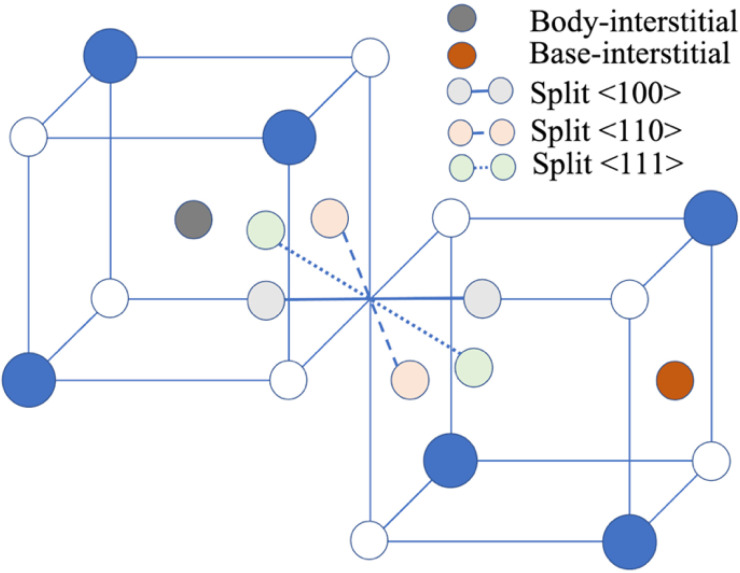
Interstitial configurations in binary rock-salt. Solid blue and open blue atoms represent cations and anions, respectively.

Five intrinsic point defects have been studied in rock-salt materials: vacancies, interstitials, and antisite defects with a single defect site and Schottky and Frenkel defects with two defect sites [13]. The vacancy and anti-site point defect sites are unique and well defined, whereas for the interstitial point defects several configurations (see Figure 1) have been explored, including the body-centered tetrahedral configuration and the split interstitials in the (110) and (111) directions.

Both types of interstitials have been reported in transition-metal nitrides [14,15,16,17] and transition-metal carbides [18,19,20], with tetrahedral sites having been considered more frequently in previous studies [21,22,23,24,25].The N <110> split interstitial is preferred over the tetrahedral interstitial sites in TiN [14,15], ScN [14], YN [14], LaN [14], VN [14], and MoN [14] and the <111> split interstitial in CrN [14] and WN [14]. In contrast, the tetrahedral site is the preferred configuration in TiC [18,20], ZrC [19], ZrN [14], HfN [14], NbN [14], and TaN [14].

Defects formed in alkali halides due to irradiation are well documented [26,27,28,29,30,31,32]. Dihalogen species, also known as H centers and with <110> orientations, are the most stable interstitial defects in alkali halides [27,28,33]. Intrinsic point defects in silver halides have been studied [34,35], including both tetrahedral and split interstitials sites for Ag, with findings indicating that split interstitials in <111> orientation are more stable for Ag_2_^+^ species.

Several theoretical studies on intrinsic point defects in lead monochalcogenides have been conducted in the last decade [21,22,36,37]. Tetrahedral interstitial defects in lead monochalcogenides have a significantly higher formation energy compared to the vacancies, and thus, Frenkel defects are less likely to form. Unfortunately, only tetrahedral sites have been considered for interstitial defects, leaving split interstitials unexplored. In addition, several theoretical studies [25,38,39] have been conducted on the intrinsic defects in alkaline-earth metal monochalcogenides (groups II–VI). In particular, Huang [25] performed a theoretical study on native defects in CaS and considered the tetrahedral site for the interstitial defect.

We recently discovered a new, mechanically stable, interstitial configuration in a study of point defects in PbS (lead sulfide) [36]—the base-centered interstitial. The body-centered (tetrahedral) interstitial is located at (0.25,0.25,0.25) and the base-centered interstitial is located at (0.25,0.25,0), defining the cubic unit cell of a rock-salt structure with eight atoms located at {(0,0,0),(0.5,0.5,0),(0.5,0,0.5),(0,0.5,0.5)} for one component and {(0.5,0,0),(0,0.5,0),(0,0,0.5),(0.5,0.5,0.5)} for the other (see Figure 1). We did not find any other stable interstitial configurations between them. Furthermore, we found that the base interstitial for sulfur (S) is preferred over the body interstitial, and an opposite result was obtained for lead (Pb) in PbS [36]. We found similar results for cation interstitials in other rock-salt materials (see Supplementary Information). Consequently, we focus on anion interstitials in rock-salts for which the base-interstitial configuration, and in some cases the split interstitial configuration, has not yet been explored.

The present study explores the stability of negative ion interstitial defects across binary rock-salt materials, including the newly discovered base-interstitial configuration. The mechanical and thermodynamic stability of several interstitial sites was examined across a range of materials, including Pb monochalcogenides, alkaline-earth metal monochalcogenides, alkali halides, and silver halides. We examined the effect of the defects on the electronic structure and conductivity of materials and the effect of charging on relative defect stability.

## 2. Methods

The Formation Energy of Interstitial Defects

Formation energy is central for determining the relative thermodynamic stability of different interstitial sites. It can be calculated as [1]

(1) $$E^{f}\left[X^{q}\right]=E_{tot}\left[X^{q}\right]-E_{tot}\left[bulk\right]-n_{i}\mu_{X}+qE_{F},$$

where $E^{f}\left[X^{q}\right]$ is the formation energy of defect X with charge q, $E_{tot}\left[X^{q}\right]$ is the total energy of a supercell containing $X^{q}$ defect after the relaxation of the ion positions, $E_{tot}\left[bulk\right]$ is the total energy of a bulk (perfect) supercell with the same number of atoms, $n_{i}$ is the number of atoms added and removed from the supercell, $\mu_{X}$ is the chemical potential of the atoms and $E_{F}$ is the Fermi energy. For a neutral defect, q equals zero in Equation (1). The formation energies of defects are for the condition (T = 0, p = 0), since the total energy is computed under this condition.

Formation energies and electronic structures of interstitial point defects in rock-salt metal monochalcogenides (e.g., Pb monochalcogenides and alkaline-earth metal monochalcogenides), alkali halides, silver halides, and TiN were calculated with supercells using the density functional theory (DFT) plane-wave pseudopotential approach. All calculations were performed using the first-principles DFT package Quantum ESPRESSO [40], with pseudopotentials obtained from the Garrity, Bennett, Rabe and Vanderbilt database [41]. The Perdew–Burke–Ernzerhof generalized gradient approximation [42] was employed for exchange and correlation energy terms without relativistic corrections.

The choice of exchange correlation functional should be motivated for the lead monochalcogenides (PbX) that present small bandgaps, which are overestimated in our calculation (GGA) (see Table 1). Incorporating the spin-orbit correction (GGA+SOC) reduces the computed bandgaps to nearly zero and predicts unphysical results [21,22]. Applying the hybrid HSE functional [43,44] leads to even larger bandgaps than with the PBE-GGA [21,22]. Finally, in an earlier study it was found that a combination of HSE and SOC leads to reasonable results, albeit at significant computational expense (see Table 2 in Ref. [22]). The suitability of our approximation (PBE-GGA) for defect electronic structure evaluations was examined in selected defects in PbS and AgBr by comparing to results from hybrid functionals with (in PbS) and without (in AgBr) relativistic corrections. We found that our method was computationally less demanding, but produced similar results for the band structure. For the purpose of the present study, accurate numerical results are not required if the qualitative picture remains the same. If required, more accurate results can be generated using hybrid functionals, including relativistic corrections for Pb compounds.

The optimized lattice parameters of the rock-salt unit cells were computationally determined. The cutoff energy of the plane-wave expansion of the wave functions was set to 40 Ry. Supercells in the present calculations were all integer multiples of the two-atom FCC primitive cell of rock-salt. The k-point sampling employed in calculations was 4 × 4 × 4 for 54-atom supercells. All the unit cell calculations were performed using a 16 × 16 × 16 k-point mesh.

Elemental phase energies were calculated for molecular nitrogen, chlorine, bromine and iodine, orthorhombic α-sulfur, and three-atom trigonal unit cells for selenium and tellurium, all of which are necessary for calculating the defect formation energies of the corresponding defects. Elemental calculations for S and Se (selenium) employed 4 × 4 × 4 and 16 × 16 × 16 k-point meshes, respectively.

## 3. Results

### 3.1. Energetic Stability of Neutral Defects

The optimized lattice constants and bandgaps of rock-salt materials were calculated, and the results are summarized in Table 1. Lattice constants were found to be in good agreement with reported experimental results. However, as expected, the DFT bandgap underestimated the experimentally determined bandgap of the materials.

#### 3.1.1. Neutral Anion Interstitial Point Defects

Anion interstitial point defects were modeled in 3 × 3 × 3 supercells of selected rock-salt materials. The defect formation energies of point defects were calculated, and the formation energies are converged to within 0.05 eV (see Table S1 in Supplementary Information). We compared the formation energies of base-, body-, and split-interstitial point defects in these materials to determine their relative stability and obtain the most stable interstitial configuration for interstitial point defects in rock-salt systems. Complementary results for cation interstitials are presented in the Supplementary Information, Table S2. Split <100> exhibited a very high formation energy in our considered systems except for AgCl, which was precluded from further consideration in this study. The results are discussed separately for Pb monochalcogenides, alkaline-earth metal monochalcogenides, alkali halides, and silver halides.

We determined that the base interstitial and split <110> were the preferred N interstitial sites in TiN, which is in agreement with a previously identified [15] split <110> interstitial site. However, because of differences in the formation energies of split <111> and body (tetrahedral) interstitials from the preferred site, split <110> or base-interstitial sites were calculated (see Table 2) and found to be 0.19 and 0.75 eV, respectively. These results are consistent with those of a previous study [15], in which the differences in the formation energies were 0.2 and 0.86 eV, respectively.

Formation energies of base-, body-, and split-interstitial point defects in lead monochalcogenides were calculated and are reported in Table 2. Formation energies of the body (tetrahedral) interstitial results in PbX are in excellent agreement with a previous computational study [22]. The defect formation energies of base-interstitial defects are lower than those of body interstitials; therefore, base-interstitial sites are preferred over body-interstitial sites. However, the lowest formation energy was found for split interstitials (split <111> in particular), which are, thus, energetically preferred over base- and body-interstitial sites for chalcogenide interstitial defects in Pb monochalcogenides materials.

To study stable interstitial sites in alkaline-earth metal monochalcogenides, we considered CaO, CaS, and MgSe and the results are summarized in Table 2. Like the Pb monochalcogenides, the base-interstitial sites had lower defect formation energies than the body interstitials. However, split <111> interstitial sites are energetically preferred in CaS and MgSe, and split <110> is preferred in CaO.

NaX, KX (X = Cl, Br, and I), RbBr, and RbI were considered to study energetically stable interstitial configurations in alkali halides. These calculated formation energies are reported in Table 2. Defect formation energies of the base interstitials in these alkali halides are lower than those of body-interstitials. Furthermore, base-interstitials are competitive with split <110> interstitials because both have nearly equal formation energies with different stable configurations after relaxation (see Section 3.1.2). Therefore, the novel base-interstitial site can be considered energetically preferred and is the ground-state interstitial site for alkali halides.

Formation energies of rock-salt silver halide interstitials were calculated and are reported in Table 2. In AgBr and AgI, the energetically preferred configurations for interstitial defects are base-centered and split <111>, respectively. Unlike the cases reported above, the defect formation energy value for the base-interstitial configuration in AgCl is larger than that obtained for the body interstitial. However, the split <111> together with split <100> interstitials have the lowest formation energies and are the preferred sites in AgCl.

The body-centered, tetrahedral structure did not have the lowest formation energy in all structures considered, although it was the most common configuration studied in literature [21,22,25,37]. Furthermore, except for AgCl, the formation energy of the body-centered interstitial was considerably higher than that of the other structures considered.

#### 3.1.2. Crystal Structure Relaxation

Defects present in the crystal displace the nearest neighboring and farther atoms from their ideal positions. The nearest neighboring atom displacements are calculated by taking the difference between their initial and final positions. Displacements for the next-nearest neighboring atoms and other atoms farther from the defect sites are considerably smaller than those of the nearest neighboring atoms. The maximum displacements of the nearest neighboring atoms for the considered defects are reported in Table S3.

We selected two materials from each family to study crystal structure relaxation due to each interstitial configuration in the rock-salt system (see Table S3). The most displaced nearest neighboring atoms for anion body-centered interstitials in rock-salt materials are anions. However, for base-centered interstitials, cations are the most displaced atoms in metal chalcogenides and potassium halides (except MgSe and KBr, for which anions are the most displaced atoms). Moreover, cations are also the most displaced atoms for split interstitials in metal chalcogenides and potassium halides. However, for silver halides, anions are the most displaced atoms in all the interstitial configurations (except split <110>, for which silver is the most displaced atom).

The results in Table S3 indicate that base-centered interstitial point defects create a larger perturbation in the crystal lattice than body-centered and split-interstitials do in metal monochalcogenides and potassium halides. A similar finding was reported in our previous work [36]. However, split interstitials create larger perturbations than base and body interstitials in silver halides.

**Figure 2:**
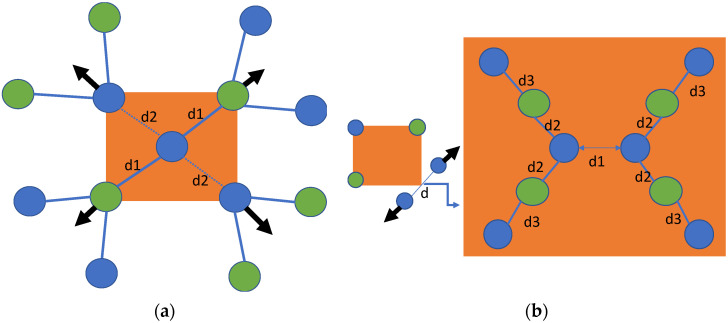
Final stable configuration of (**a**) base and (**b**) split <110> interstitials in alkali halides. Blue and green atoms are halogens and alkali metals, respectively. Arrows represent the direction of motion and their magnitude represents relative displacements corresponding to nearest neighboring atoms.

The formation energy results in Table 2 show that the base and split <110> configurations have nearly equal formation energies in alkali halides and are equally energetically favored. Therefore, we analyzed the displacements of the nearest neighboring atoms in the base-centered interstitial and split <110> point defects in detail and found that they were stabilized in different configurations after relaxation, as shown in Figure 2. The nearest neighboring atoms were displaced on the same (110) plane and in an outward direction (see Figure 2a). Because of the displacement of the nearest neighboring atoms, they interact with the next-nearest atoms (see Figure 2a). Halogens present at the base-interstitial site have a strong interaction with neighboring alkali atoms and a weaker interaction with the nearest halogens (i.e., $d_{2}>d_{1}$, according to the notation in Figure 2; see Figure 2a). Split <110> halogen atoms are displaced in an outward direction ($d_{1}>d,$ see Figure 2b), and each halogen interacts with two nearest neighboring alkali atoms (see Figure 2b). Consequently, these alkali atoms are also displaced from their ideal positions and have a relatively weaker interaction with a halogen atom (next-nearest atom) in the stable, relaxed configuration, as shown in Figure 2b ($d_{2}>d_{1}>d_{3}$). Similar results were found for base-interstitial defects in PbS [36].

### 3.2. Bandgap and Electronic Properties of Neutral Defects

**Figure 3:**
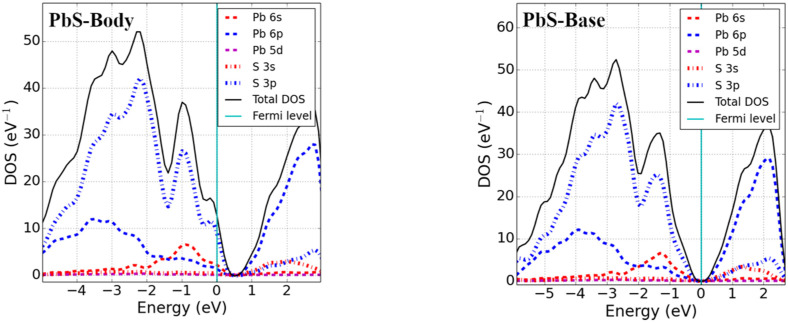

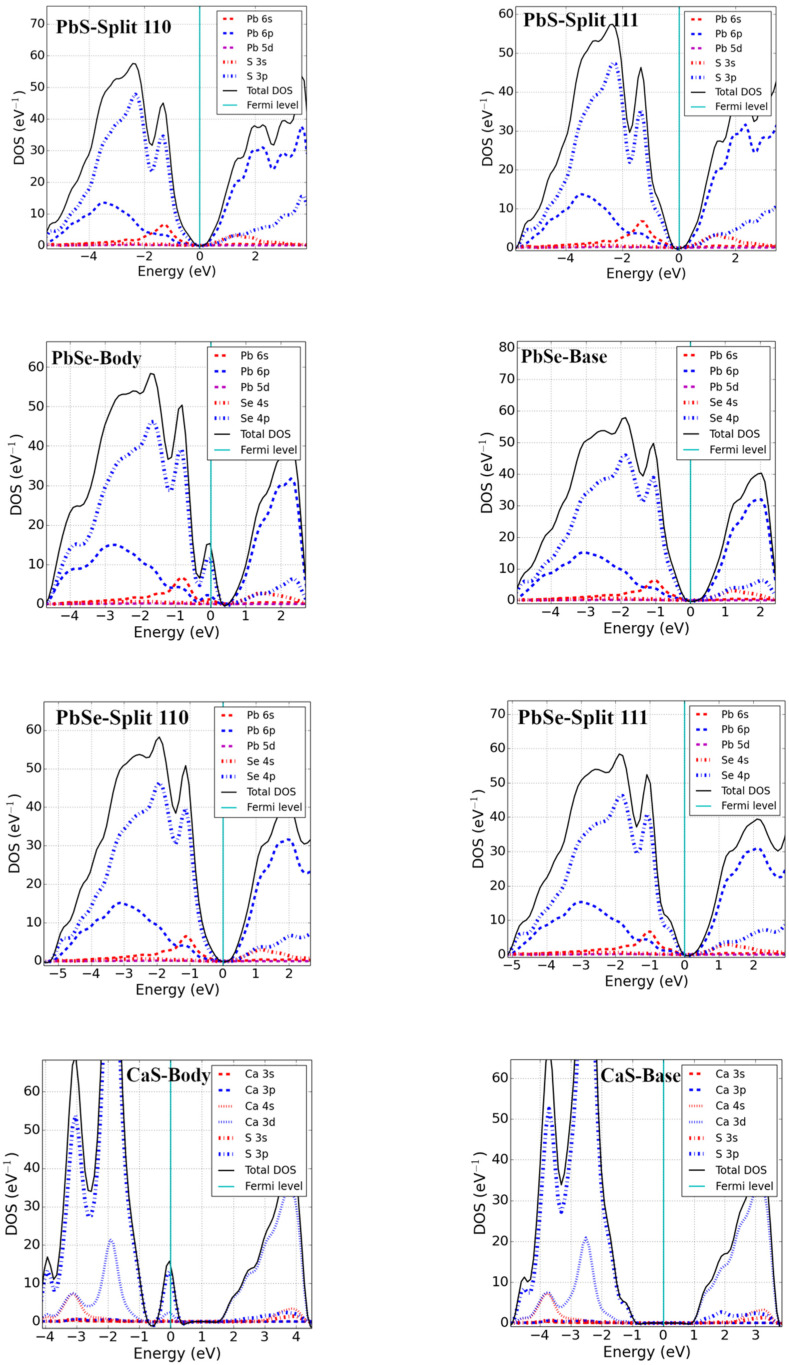

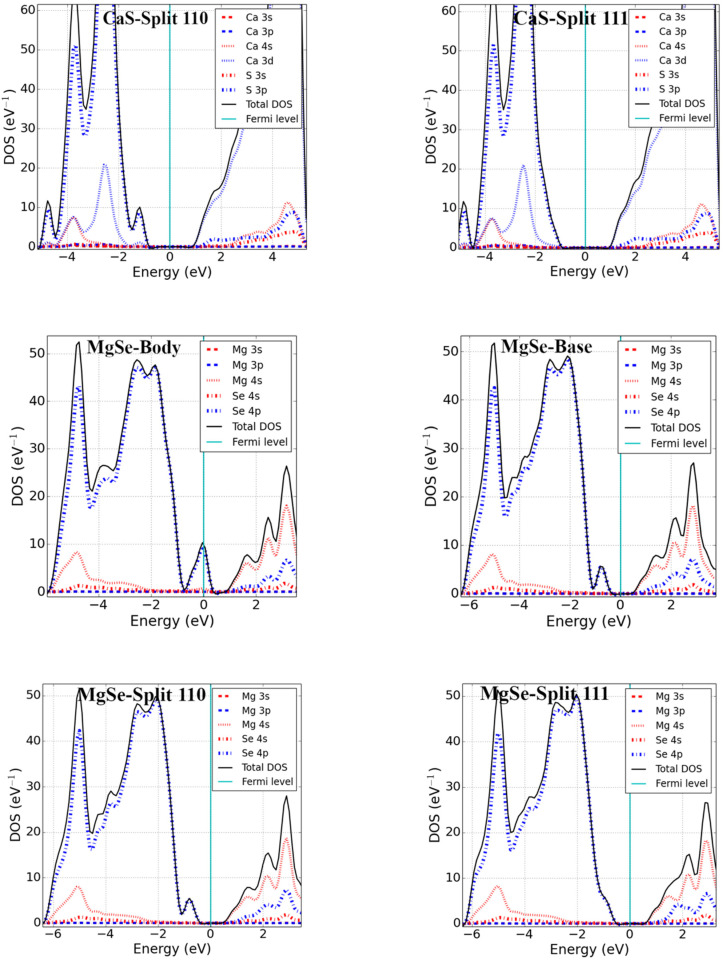

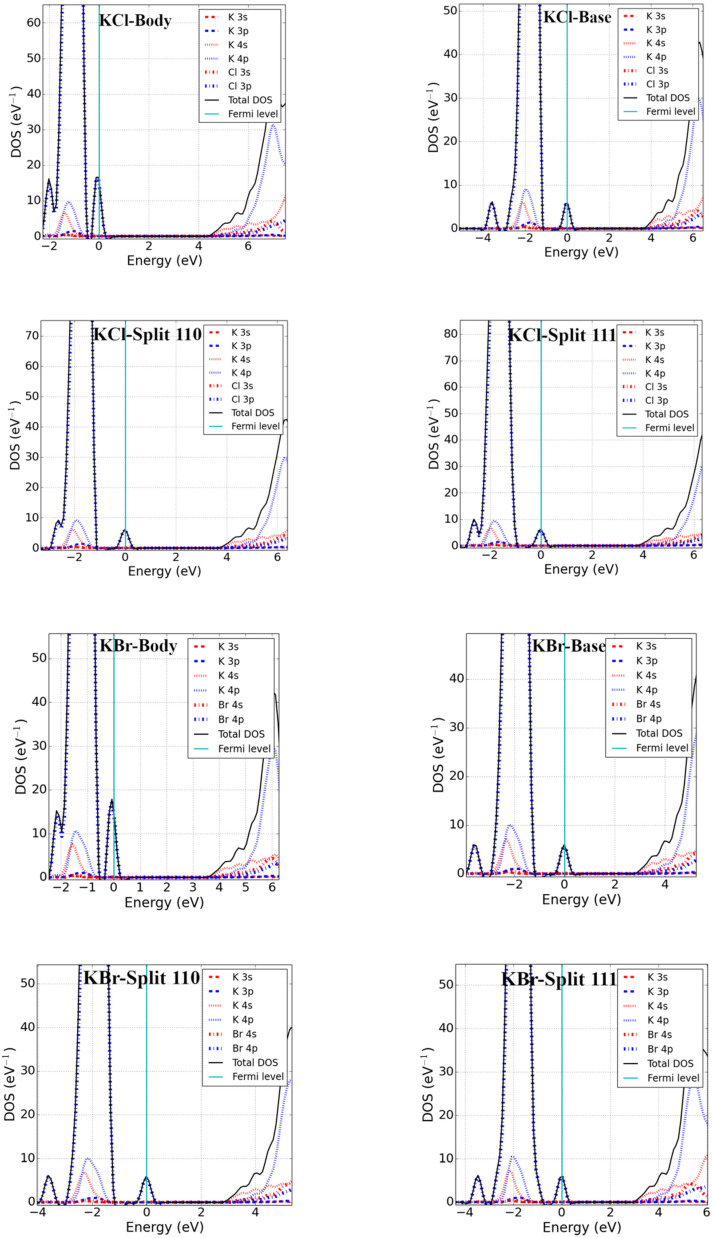

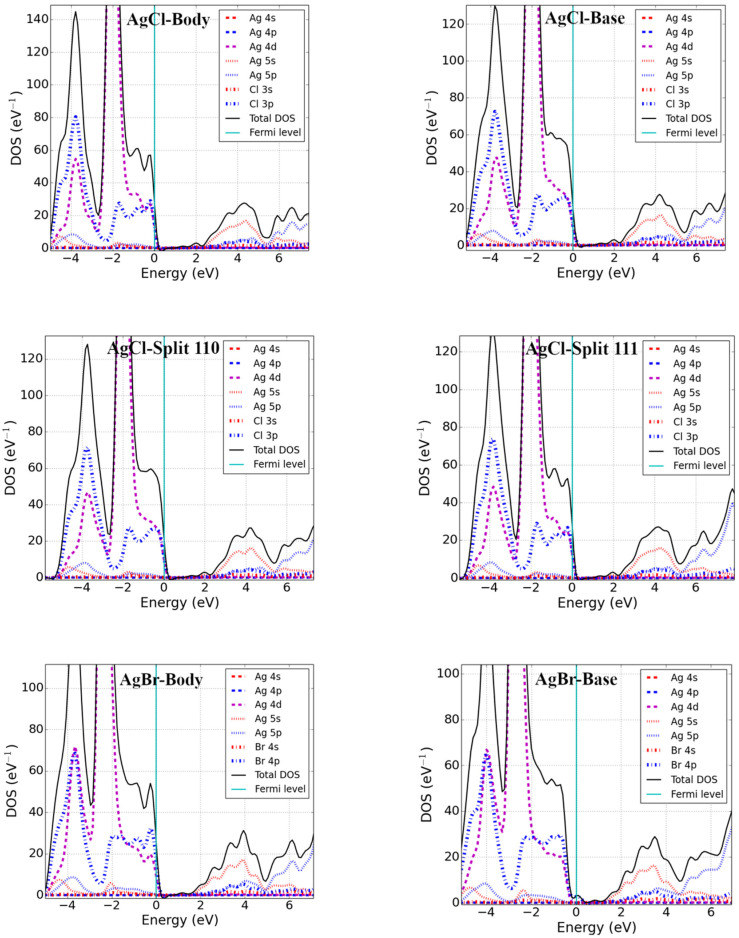

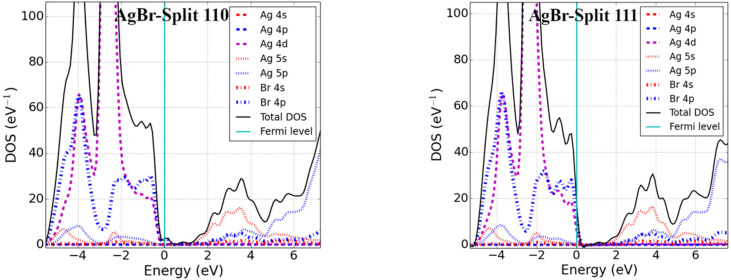
Total DOS and pDOS of 3 × 3 × 3 supercells with interstitial point defects (Fermi level set to zero).

Point defects may change the electronic properties of pristine materials by perturbing the valence band maxima (VBM) and conduction band minima (CBM). We studied the electronic properties of interstitial point defects in selected rock-salt materials, examining two materials from each family as noted in Section 3.1.2. Total density of states (DOS) and projected density of states (pDOS) calculations were performed using the optimized structure of rock-salt materials and a Fermi level set to zero, which is graphically represented in Figure 3.

The addition of an interstitial atom introduces partially filled p-states into the band structure. These can be localized in the bandgap, as we found for body-centered interstitials in PbSe, CaS, and MgSe as well as potassium halide interstitials (see Figure 3). Alternatively, the additional states could be located below the VBM-supporting p-type conductivity in the defective systems. Examining the different rock-salt material families in detail, we found the following:

(i) Pb monochalcogenides (PbX; X = S, Se)

For Pb monochalcogenides, the Fermi level was found to lie near the top of the valence band within the continuous DOS for an X body-interstitial, and no fundamental bandgap was formed. The defect states were found near the VBM and were unoccupied, with the p-state of X slightly mixed with the s-state of Pb. Therefore, given the condition of excess body-interstitial point defects in Pb monochalcogenides, the system is expected to behave as p-type. For X base-interstitial defects, a splitting of states near the VBM was seen in the band structure, which produced a fundamental band gap equal to that of an ideal supercell. Essentially, the excess empty S p-state hybridized with the Pb p-states and shifted to the conduction band, as observed from the DOS. The electronic structure of split interstitials showed a behavior similar to that of base-interstitial defects. No significant change in DOS near the VBM and CBM was observed; however, an increased DOS deep in the conduction band could be perceived.

(ii) Alkaline-earth metal monochalcogenides (MX; M = Ca, Mg; X = S, Se)

The DOS calculations of alkaline-earth metal monochalcogenides revealed that the VBM of MX was mainly due to the p-state of X and the CBM was due to the p-states of M. Unlike the body-interstitials of lead monochalcogenides, a localized defect state was obtained above the VBM in MgSe and CaS. The p-state of Se formed the localized defect state in MgSe and the p-state of S was slightly mixed with the d-state of Ca in CaS, enabling it to act as a strong acceptor center. The result is consistent with a previous theoretical study on CaS [25]. However, like base-interstitial in Pb monochalcogenides, the Fermi level shifted inside the bandgap and opened the fundamental bandgap for base-interstitials in MgSe and CaS. Split-interstitials showed an electronic structure similar to the base interstitials.

(iii) Potassium halides (KX′; X′ = Cl, Br)

The DOS of potassium halides were calculated (presented graphically in Figure 3) and indicated that all interstitial configurations showed nearly similar electronic structures. The VBM of KX’ was largely due to the p-states of X’. However, the CBM was formed from the mixed s and p of K. Just as with the body-interstitial defects in alkaline-earth metal monochalcogenides, a localized defect state was obtained inside the bandgap (near the VBM) for all interstitial defects, and these defects closed the fundamental bandgap of pristine materials in potassium halides. From the pDOS calculation, the defect states were due to the p-states of halides. However, the electronic structures of the split-interstitials were nearly identical to those of the base-interstitials.

(iv) Silver halides (AgX′; X′ = Cl, Br)

DOS calculations were performed for silver halide (AgCl and AgBr) materials, and findings showed that the CBM was predominately formed from the 5s-states of Ag, while the VBM was due to the mixing of 4d of Ag and 3p (4p) of Cl (Br) for AgCl (AgBr). The Fermi level was found to lie near the VBM within the continuous DOS of the valence band for the body-, base-, and split-interstitials in AgCl. In addition, a similar result was obtained for the body and split <111> interstitials in AgBr. However, like the interstitial defects in potassium halides, a localized defect state was located just above the VBM for the base and split <110> defects. Defect states of the body and split <111> interstitials were formed from 3p (4p) of Cl (Br) and overlapped with 4d of Ag for AgCl (AgBr). Similar results can be seen for the base and split <110> interstitials in AgCl; however, their defect states were due to the 4p of Br in AgBr.

### 3.3. Investigation of Charged Interstitial Defects

The relative thermodynamic stability of neutral interstitial configurations in rock-salt materials was examined in Section 3.1.1 (see Table 2). However, varying the potential can cause the relative stability of charged defects to vary. The formation energies of charged interstitials in the body-centered, base-centered and split configurations were calculated using Equation (1) for selected rock-salt materials. The Fermi energy (in the last term of Equation (1)) can be expanded into two terms $\left(E_{f}=E_{VBM}+\Delta E_{f}\right)$; for insulators and semiconductors, it is assumed to vary between the VBM and the CBM. Because we studied only interstitial defects in the present work, we ignored the effect of chemical potentials. Our calculations were performed with reference to the formation energy of the anions in their elemental phase (conventional chemical potential). The results are discussed separately for different classes of rock-salt materials.

(i) Pb monochalcogenides

**Figure 4:**
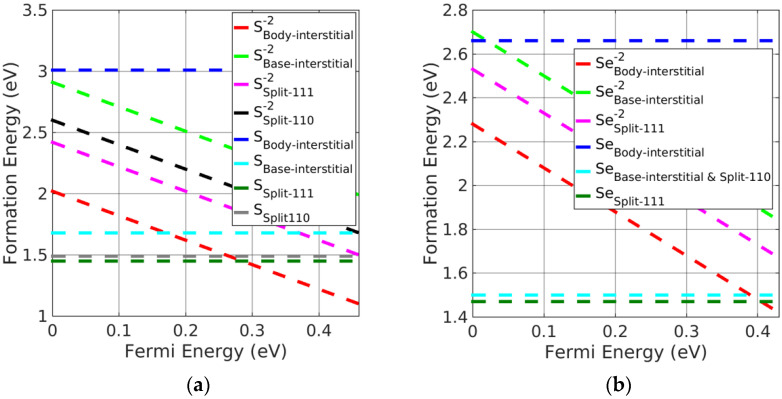
Formation energy of charged interstitial defects in (**a**) PbS at S−rich and (**b**) PbSe at Se−rich conditions.

Split-interstitials were found to be the stable neutral interstitials in Pb monochalcogenides. The stability of all the interstitial defects was investigated based on the calculated formation energies. We found that the base-interstitials in the neutral state were preferred over doubly charged states, where body-interstitials were preferred. The order of stability for the charged interstitials in PbSe was as follows: body-interstitial > (split <111>) > base-interstitial > (split <110>); this is shown in Figure 4b, excluding charged split <110>, which is the least-stable interstitial defect. For PbS, the trend was similar except for split <110>, which was greater than the base-interstitial. Moreover, neutral split <111> was the preferred interstitial site followed by split <110> and base-centered interstitials in Pb monochalcogenides. However, doubly charged body-interstitial defects become favorable when the Fermi energy approaches the CBM. It can be seen from Figure 4a,b, that a neutral split <111> interstitial was the most likely defect to form in PbX.

(ii) Alkaline-earth metal monochalcogenides

**Figure 5:**
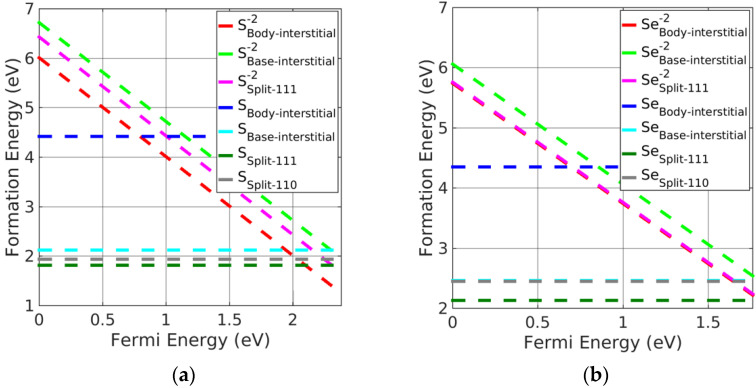
Formation energy of charged interstitial defects in (**a**) CaS at S−rich and (**b**) MgSe at Se−rich conditions.

As with Pb monochalcogenides, the split-interstitial defects were stable in a neutral charge state (see Figure 5a,b). Nevertheless, body-interstitial defects were stable in their doubly charged state. Similar to PbSe, the neutral split <111> interstitials were the preferred interstitial site, followed by split <110> and base-interstitials; however, charged body-interstitial defects were preferred for a Fermi energy close to the CBM. The stability order of charged interstitial defects in alkaline-earth metal monochalcogenides was similar to the order obtained in PbSe.

(iii) Potassium halides

**Figure 6:**
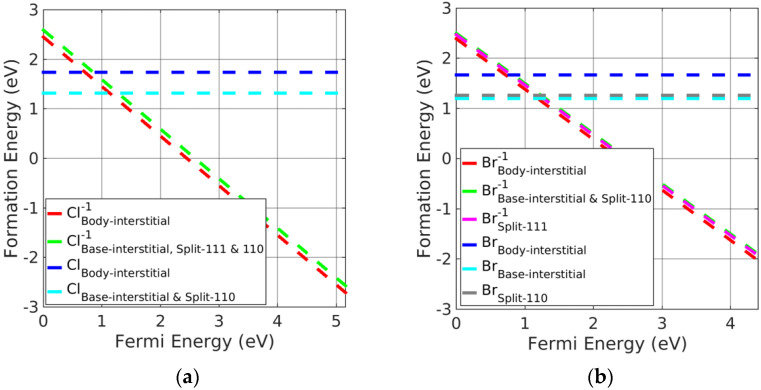
Formation energy of charged interstitial defects in (**a**) KCl at Cl−rich and (**b**) KBr in Br−rich conditions.

Potassium halide charged-interstitial point defects are presented in Figure 6a,b. Neutral base-interstitial and split <110> point defects had greater stability than the neutral split <111> and body-interstitials. However, charged body-interstitial defects were slightly preferred over other charged interstitial defects. Figure 6 indicates that the neutral base-interstitial and split <110> point defects were the preferred interstitial point defects when the Fermi energy was near the VBM. However, singly charged body-interstitials followed by rest charged interstitials are otherwise stable. In addition, all the interstitial defects change their neutral charge states to singly charged states near the VBM. Therefore, charged interstitial defects are stable defects in potassium halides.

(iv) Silver halides

**Figure 7:**
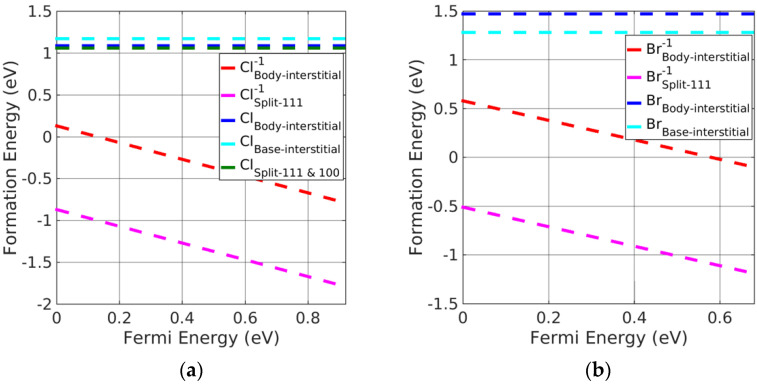
Formation energy of charged interstitial defects in (**a**) AgCl at Cl−rich and (**b**) AgBr at Br−rich conditions.

The charged split <111> interstitial point defects had significantly lower formation energy, followed by charged body interstitials, compared with the neutral interstitial defects, making the former the preferential defects in silver halides. However, charged split <110> and base-interstitials showed significantly higher formation energies (and therefore, values are not included in the plots of Figure 7a,b) in silver halides and were barely formed. In addition, split <100> and split <111> interstitials were found to be leading interstitials for neutral interstitial defects in AgCl; however, charged split <100> interstitials showed significantly larger formation energy than split <111> interstitials (values are not added in the plot of Figure 7a,b). Thus, the charged split <111> interstitial point defects are energetically favorable in silver halide rock-salt materials.

## 4. Discussion

The stability of anion interstitial point defects in rock-salt AB material systems was studied and the defect formation energies were calculated with reference to conventional chemical potentials. Because we performed a comparative study of interstitial point defects, the chemical potential effects were not critical in this work. We found that the newly discovered base-interstitials were stable and competitive sites in rock-salt materials. They were also the ground states of interstitials in several rock-salt materials, e.g., alkali halides.

In the relaxation process of the base-interstitial configuration, the nearest neighboring atoms were displaced outwards on the same plane, i.e., (110). Generally, base configurations create larger perturbations in the crystal lattice than body interstitials. Base configurations and split <110> interstitials are also stable configurations in alkali halides. However, both are stabilized in different relaxed configurations (see Figure 2). We obtained the optimal Cl–Cl distance for split <110> in KCl (2.55 Å), and our results are in good agreement with earlier reported theoretical values (e.g., 2.52 [30] and 2.60 Å [28]).

For neutral interstitial defects, split-interstitial defects are energetically favored defects over base- and body-interstitials in metal monochalcogenides and silver halides, except for AgBr. Unfortunately, these configurations have been ignored in many previous studies of interstitials in rock-salt materials, e.g., Refs. [21,22,25,37] Split-interstitials are the preferred configurations in metal monochalcogenides. However, silver halide systems have distinct energetically stable interstitial preferences as the halide changes. In addition, we found that base interstitials were competitive with split-interstitial sites in several rock-salt materials (e.g., lead selenides and alkali halides). However, the base-interstitial is relatively stable compared to the body- and split-interstitials in AgBr. Moreover, base-interstitial defects were energetically more favorable than the most explored body-interstitials in rock-salt materials. Therefore, the most stable sites for anion interstitial point defects in rock-salt materials are either base- or split-interstitials.

We explored the charge stability of interstitial defects and found that neutral base-interstitial defects are preferred over doubly charged defects in metal monochalcogenides and silver halides. However, base-interstitial defects are stable in a doubly charged state for alkali halides. In addition, neutral base-interstitials and split-interstitials are energetically favored over doubly charged body-interstitials in metal monochalcogenides. However, charged body-interstitial defects are energetically more stable than the neutral interstitial defects in potassium halides. Charged split <111> interstitial defects are energetically favorable interstitial defects in silver halides.

The interstitial site contributes to the formation of composite defects (e.g., Frenkel defects), in which an atom vacates its site to form a vacancy and occupies the interstitial site. Because of the availability of several interstitial sites in rock-salt materials, several respective types of Frenkel defects may form. In our previous study, we investigated two Frenkel defects due to base- and body-interstitial occupancies in PbS [36]. In addition, point defects are used to store energy in materials [3], and these Frenkel defects must be considered in the study.

The electronic band structure of ideal rock-salt materials is strongly affected by the body-, base-, and split-interstitials. In particular, the bandgap was discovered to be a sensitive measure of variations in electronic properties. Chalcogen body-interstitial sites close the fundamental band gap and Fermi level found inside the continuous DOS of the valence band for Pb monochalcogenides. The defect states are made up of chalcogen p-states and are unoccupied, causing materials to become p-type conductors. Similar behavior can be seen because of metal vacancies in Pb monochalcogenides [22,36]. However, a localized defect state was introduced inside the bandgap for a body-interstitial defect in alkaline-earth metal monochalcogenides. These results are consistent with those of a previous study on CaS [25]. In addition, the Fermi level was found inside the bandgap for the base- and split-interstitials, opening the fundamental band gap (i.e., nearly equal to the ideal material bandgap due to the hybridization of the p-states of chalcogens with excess p-states of metals in metal monochalcogenides). Nevertheless, body-, base-, and split-interstitials induce a localized defect state near the VBM in alkali halides. In addition, similar to the body-interstitial defects in metal monochalcogenides, in all interstitial defects of AgCl, the Fermi level is found inside the continuous DOS of the valence band near the VBM forming a p-type conductor. A similar electronic structure is obtained for body and split <111> interstitials in AgBr. However, the electronic structures of base and split <110> in AgBr are similar to the interstitial defects in alkali halides.

**Figure 8:**
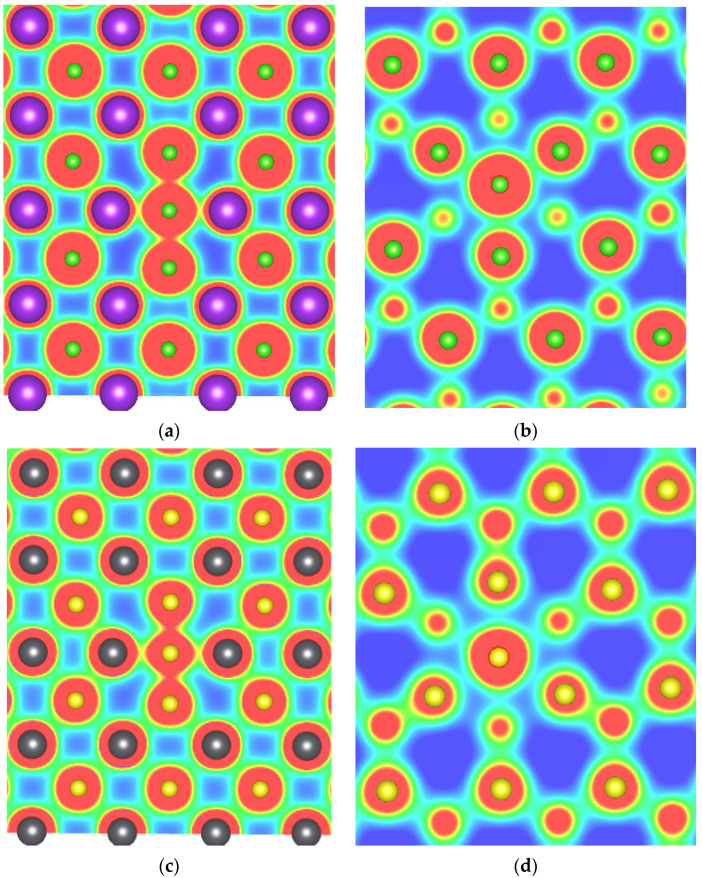

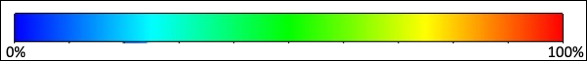
Charge density of (**a**) Cl base, (**b**) body-interstitial in KCl, (**c**) S base and (**d**) body-interstitial in PbS. Green, purple, black, and yellow atoms represent chlorine, potassium, lead, and sulfur atoms, respectively.

Charge density calculations were performed to understand the bonding between anions at base or body interstitial sites with their neighboring atoms (see Figure 8). We consider here two materials, KCl and PbS, from the alkali halide and lead monochalcogenide families, respectively. From Figure 8a,c, it can be observed that anions at base interstitial sites show stronger interaction with neighboring anions than with neighboring cations, indicating the formation of bonds between them. Furthermore, anions at body interstitial sites showed relatively stronger interaction with nearest anions than the cations in KCl; however, interactions are nearly equal with nearest cations and anions in PbS. Thus, we conclude that anions at the base interstitial site bond with two nearest neighbouring anions and form a $X_{3}^{q}$ entity that stabilizes this defect in binary rock-salt systems.

## 5. Conclusions

In the rock-salt system, the novel base-interstitial sites are mechanically and energetically stable sites for interstitial point defects. These sites are energetically preferred sites for anion interstitial point defects in several rock-salt materials, e.g., alkali halides, due to formation of a $X_{3}^{2-}$ (X = Cl, Br, I) entity. We expect that the discovery of base interstitials may modify our understanding of anion migration in binary rock-salt systems.

## Figures and Tables

**Table 1:** Calculated lattice constants (in Å) and bandgaps (in eV) of optimized unit cells for selected rock-salt materials.

Materials	Lattice Constant (Å)	Experimental Lattice Constant (Å)	Bandgap (eV)	Experimental Bandgap at 300 K (eV)
PbS	5.981	5.936 [45]	0.46	0.41 [46]
PbSe	6.215	6.124 [45]	0.43	0.29 [46]
PbTe	6.562	6.462 [45]	0.80	0.32 [46]
CaO	4.831	4.811 [45]	3.67	7.70 [47]
CaS	5.71	5.69 [48]	2.38	5.80 [46]
MgSe	5.508	5.46 [45]	1.76	5.6 [46]
NaCl	5.705	5.64 [49]	4.98	8.75 [49]
NaBr	6.039	5.977 [49]	4.06	7.10 [49]
NaI	6.533	6.473 [49]	3.57	5.90 [50]
KCl	6.308	6.293 [49]	5.16	8.4 [49]
KBr	6.626	6.597 [49]	4.40	7.4 [49]
KI	7.084	7.066 [49]	3.95	6.0 [49]
RbBr	7.012	6.889 [49]	4.20	7.5 [49]
RbI	7.487	7.342 [49]	3.77	6.2 [49]
AgCl	5.581	5.55 [51]	0.92	3.25 [52]
AgBr	5.812	5.77 [34]	0.68	2.68 [52]
AgI	6.128	6.07 [51]	0.68	2.33 [52]
TiN	4.247	4.25 [53]		

**Table 2:** Formation energies (eV) of anion interstitial configurations in rock-salt systems. The configuration with the lowest energy in each system is marked in bold.

Lead Monochalcogenides			
Defect	PbS	PbSe	PbTe
	Formation energy	Formation energy	Formation energy
Base-interstitial	1.68	1.5	1.82
Body-interstitial	3.01	2.66	2.69
Split <111>	**1.45**	**1.47**	**1.76**
Split <110>	1.49	1.50	1.82
Split <100>	2.13	2.39	3.14
**Alkaline-Earth Metal Monochalcogenides**			
	MgSe	CaO	CaS
	Formation energy	Formation energy	Formation energy
Base-interstitial	2.46	2.04	2.12
Body-interstitial	4.35	3.56	4.42
Split <111>	**2.13**	1.31	**1.82**
Split <110>	2.45	**1.18**	1.94
Split <100>	4.82		2.93
**Potassium Halides**			
	KCl	KBr	KI
	Formation energy	Formation energy	Formation energy
Base-interstitial	**1.32**	**1.2**	**1.04**
Body-interstitial	1.74	1.67	1.55
Split <111>	1.41	1.31	1.15
Split <110>	**1.32**	1.26	1.06
Split <100>	1.73		
**Sodium Halides**			
	NaCl	NaBr	NaI
	Formation energy	Formation energy	Formation energy
base-interstitial	**1.71**	**1.55**	**1.41**
body-interstitial	2.3	2.16	2.01
split <111>	1.82	1.69	1.55
split <110>	**1.71**	1.56	**1.41**
split <100>	2.38	2.28	
**Rubidium Halides**			
	RbBr	RbI	
	Formation energy	Formation energy	
Base-interstitial	**1.03**	**0.90**	
Body-interstitial	1.43	1.34	
Split <111>	1.13	0.99	
Split <110>	1.05	0.91	
Split <100>		1.37	
**Silver Halides**			
	AgCl	AgBr	AgI
	Formation energy	Formation energy	Formation energy
Base-interstitial	1.15	**1.28**	1.33
Body-interstitial	1.09	1.47	1.75
Split <111>	**1.06**	1.31	**1.11**
Split <110>	1.21	1.34	1.34
Split <100>	**1.06**	1.49	1.79
**Transition-Metal Nitride**			
	TiN		
	Formation energy		
Base-interstitial	**4.08**		
Body-interstitial	4.83		
Split <111>	4.27		
Split <110>	**4.08**		
Split <100>	4.98		

## Data Availability

The raw/processed data required to reproduce these findings cannot be shared at this time, as the data also form part of an ongoing study.

## Supplementary Materials

The following supporting information can be downloaded at: https://www.mdpi.com/article/10.3390/ma15176015/s1, Table S1: Defect formation energy (eV) of selected defects with supercell size. The formation energies are converged to better than 0.05 eV with the supercell size; Table S2: Defect formation energy (eV) of cation interstitials in selected rock salt materials. Defect formation energy of the most stable interstitial configuration (body interstitial) is set to zero; Table S3: Displacement (in Å) of the nearest-neighboring atoms from their original positions in the defect-bearing supercells. The most displaced configuration in each system is marked in bold.
