# Supplementary material for: A Novel Interstitial Site in Binary Rock-Salt Compounds
Source: Materials (Basel). 2022 Aug 31;15(17):6015. doi: 10.3390/ma15176015 (PMC9457209; doi:10.3390/ma15176015)
Supplement: Supplementary file 1 [file materials-15-06015-s001.zip › materials-1861100-supplementary.pdf]

Supplementary Information

A novel interstitial site in binary rock-salt compounds

Neeraj Mishra and Guy Makov

Dept. of Materials Engineering, Ben-Gurion University of the Negev, Beer Sheva 84105, Israel

Materials	Defects	Formation energy (eV) (3x3x3)	Formation energy (eV) (4x4x4)
KCl	Cl- body interstitial	1.74	1.75
	Cl- base interstitial	1.32	1.31
	Split <111>	1.41	1.40
	Split <110>	1.32	1.32
PbS	S- body interstitial	3.01	3.06
	S- base interstitial	1.68	1.69
	S- vacancy	2.05	2.05
	Split <111>	1.45	1.43
	Split <110>	1.49	1.52
AgBr	Br- Split <111>	1.31	1.36

Table S1. Defect formation energy (eV) of selected defects with supercell size. The formation energies are converged to better than 0.05 eV with the supercell size.

Materials	Defects	Formation energy<br>(eV)<br>(3x3x3)
KCl	K- body interstitial<br>K- base interstitial	0<br>0.2
KBr	K- body interstitial<br>K- base interstitial	0<br>0.2
PbS	Pb- body interstitial<br>Pb- base interstitial	0<br>0.40
AgCl	Ag- body interstitial<br>Ag- base interstitial	0<br>0.06

Table S2. Defect formation energy (eV) of cation interstitials in selected rock salt materials. Defect formation energy of the most stable interstitial configuration (body interstitial) is set to zero.

**Materials**	**Defects**	**Most displaced atom**	**Displacements (Å)**
PbS	Body	S	0.40
	**Base**	Pb	0.58
	Split <111>	Pb	0.38
	Split <110>	Pb	0.43
PbSe	Body	Se	0.37
	**Base**	Pb	0.64
	Split <111>	Pb	0.45
	Split <110>	Pb	0.49
CaS	Body	S	0.36
	**Base**	Ca	0.52
	Split <111>	Ca	0.37
	Split <110>	Ca	0.44
MgSe	Body	Se	0.54
	**Base**	Se	0.63
	Split <111>	Mg	0.52
	Split <110>	Mg	0.53
KCl	Body	Cl	0.42
	**Base**	K	0.61

	Split <111>	K	0.32
	Split <110>	K	0.50
KBr	Body	Br	0.48
	**Base**	Br	0.69
	Split <111>	K	0.37
	Split <110>	K	0.54
AgCl	Body	Cl	0.69
	Base	Cl	0.99
	Split <111>	Cl	1.02
	**Split <110>**	Ag	1.20
AgBr	Body	Br	0.71
	Base	Br	0.98
	**Split <111>**	Br	0.99
	Split <110>	Ag	0.83

Table S3. Displacement (in Å) of the nearest-neighboring atoms from their original positions in the defect-bearing supercells. The most displaced configuration in each system is marked in bold.
